# B‐Type Natriuretic Peptides Levels in Patients With Beta‐Thalassemia Major and Correlations With Biomarkers: A Systematic Review and Meta‐Analysis

**DOI:** 10.1002/hsr2.71493

**Published:** 2025-11-09

**Authors:** Mohamed S. K. Salih, Amna H. Mohamed, Elsara M. A. Mirghani, Mohammed Y. K. Makki, Ola A. M. Ahmed, Esraa T. S. Mohammed, Hana H.M. Hassan, Nada Omar, Esraa M. A. Garalnabi, Sagad O. O. Mohamed

**Affiliations:** ^1^ Harlem Hospital Center New York New York USA; ^2^ University of Khartoum Khartoum Khartoum Sudan; ^3^ Alzaiem Alazhari University Khartoum Sudan; ^4^ Alneelain University Khartoum Sudan; ^5^ Newcastle Upon Tyne NHS trust UK

**Keywords:** Beta‐thalassemia, BNP, B‐type natriuretic peptides, NT‐proBNP, systematic review

## Abstract

**Background:**

Patients with beta‐thalassemia major are prone for developing cardiovascular complications. Early identification of patients at risk is important. B‐type natriuretic peptides such as brain natriuretic peptide (BNP) and N‐terminal pro‐BNP (NT‐proBNP) are sensitive indicators of cardiac dysfunction. This review synthesizes the existing evidence on the levels of B‐type natriuretic peptides in beta‐thalassemia major and their relationship with markers of cardiac dysfunction and other biomarkers.

**Methods:**

A systematic review was conducted according to PRISMA guidelines. The search was performed in the PubMed, Web of Science, ScienceDirect, and World Health Organization Virtual Health Library Regional Portal. Pooled standardized mean differences (SMD) and correlation coefficients (*r*) with 95% confidence intervals (CI) were calculated to investigate the differences in B‐type natriuretic peptides values and their relationships with various biomarkers in beta‐thalassemia major.

**Results:**

A total of 29 studies were selected for the systematic review. Of them, 27 were included in the meta‐analyses. Most of the included studies focused on investigating NT‐proBNP. The analyses revealed significantly higher NT‐proBNP and BNP values in patients with beta‐thalassemia major compared to healthy controls. For NT‐proBNP, the pooled effect size (SMD) was 1.37 (95% CI: 0.856–1.893, *p* < 0.001), and for BNP, it was 1.94 (95% CI: 0.665–3.220, *p* = 0.003). Meta‐analyses of correlations revealed that NT‐proBNP was significantly correlated with serum ferritin levels (*r* = 0.471, *p* = 0.006), E/E′ ratio (*r* = 0.528, *p* < 0.001), and patients age (*r* = 0.259, *p* = 0.004).

**Conclusion:**

This systematic review provides evidence that beta‐thalassemia is associated with high levels of B‐type natriuretic peptides, which highlight the high risk of cardiovascular complications in this patient population. The significantly elevated levels of both molecules and their identified correlations emphasize clinical utility of them as markers of early myocardial stress and subclinical heart disease in beta‐thalassemia.

**Abbreviations**
Brain Natriuretic Peptide: (BNP)
Cardiac T2* magnetic resonance imaging: (MRI‐T2)
Early trans‐mitral inflow velocity: (E)
Late trans‐mitral inflow velocity: (A)
Mitral annular velocity in early diastole: (E′)
Mitral annular velocity in late diastole: (A′)
N‐terminal proBNP: (NT‐proBNP)

## 1 Background

Beta‐thalassemia is a prevalent inherited blood disorder affecting approximately 1.5% of the global population [1]. It is particularly common in the Mediterranean, North Africa, and Central Africa, but due to increased migration, its incidence has significantly expanded into other areas, including Northern Europe and North America [2]. This genetic disorder results from mutations in the beta‐globin gene, leading to reduced or absent production of beta‐globin chains, which are essential components of hemoglobin [1, 2, 3, 4]. The defective hemoglobin function disrupts erythropoiesis, causing severe chronic anemia and a spectrum of clinical manifestations. Based on genetic defects and disease severity, beta‐thalassemia is classified into three major categories: beta‐thalassemia major, thalassemia intermedia, and thalassemia minor [2, 3]

A major complication of the disease is iron overload due to repeated blood transfusions, leading to iron deposition in various organs, including the liver, endocrine glands, and heart [5]. Among these, cardiac complications are the most severe and life‐threatening, with iron‐induced cardiomyopathy being a leading cause of mortality in this patient population [6]. With repeated blood transfusions, iron accumulates in the heart, increasing the risk of heart failure and arrhythmias [6]. Iron chelation therapy is crucial for mitigating these complications, but early and reliable markers of cardiac dysfunction are essential for timely intervention [7, 8]

While cardiac T2* magnetic resonance imaging (MRI‐T2) can detect iron‐related heart complications early, its practical limitations regarding high costs and lengthy scan times highlight the need for more practical and accessible biomarkers [9, 10]. Finding alternative diagnostic tools that are both practical and widely available would significantly improve the monitoring and management of these cardiac complications.

B‐type natriuretic peptides (Brain Natriuretic Peptide [BNP], ProBNP, and N‐terminal proBNP [NT‐proBNP]), are secreted by cardiomyocytes in response to ventricular wall stress and have been investigated as potential early indicators of cardiac dysfunction [11]. They serve as crucial biomarker for investigating both symptomatic and asymptomatic heart dysfunction and their levels correlate significantly with the severity of cardiac conditions, making them valuable tools in clinical settings [12]. Several studies have explored the correlation between B‐type natriuretic peptides levels and echocardiographic markers of cardiac dysfunction in the general population. However, findings from studies on beta‐thalassemia populations are limited and have been inconsistent across studies. Their clinical application in beta‐thalassemia is currently hampered by significant inconsistencies across published studies. Individual studies report conflicting findings on the strength of the correlation between NT‐proBNP and key cardiac parameters, and propose a wide range of diagnostic cut‐off values, making it difficult for clinicians to interpret results in a standardized way. This lack of consensus is a barrier to the development of evidence‐based clinical guidelines for cardiac monitoring in beta‐thalassemia population.

Therefore, this systematic review and meta‐analysis aims to address this study gap. We systematic review aims to synthesize available evidence and provide a comprehensive, generalizable estimate of the correlation between B‐type natriuretic peptides levels and markers of cardiac dysfunction in beta‐thalassemia patients, thereby contributing to improved clinical management and patient outcomes in this vulnerable patient population.

## 2 Methods

Search approach and studies inclusion criteria:

This review followed the guidelines outlined in the Preferred Reporting Items for Systematic Reviews and Meta‐Analyses (PRISMA) statement [13]. The systematic review was previously registered on the Open Science Framework platform (https://osf.io/zey9v). To identify relevant literature, an electronic literature search was conducted via the databases of PubMed, Web of Science, ScienceDirect, and World Health Organization Virtual Health Library Regional Portal. The search strategy did not impose any limitations based on geographical location or publication date, aiming to capture the widest possible range of relevant studies.

We sought to determine whether there is a significant difference in the levels of B‐type natriuretic peptides (BNP, ProBNP, and NT‐proBNP) between beta‐thalassemia patients and healthy subjects, and to investigate the relationship between these peptides and any identified markers of cardiac dysfunction in beta‐thalassemia patients. Therefore, PECO and PEO models were used to frame the following research questions:

Primary research question: in patients with beta‐thalassemia (Population), how does the presence of beta‐thalassemia (Exposure) compared to healthy individuals (Comparator) affect the levels of natriuretic peptides (Outcome)? Secondary research question: in patients with beta‐thalassemia (Population), how do varying levels of natriuretic peptides (Exposure) correlate with any identified markers of cardiac dysfunction (Outcome)?

The electronic search was conducted on literature from inception up to December 2024, using the following key words: (thalassemia OR “beta‐thalassemia” OR β‐thalassemia) AND (Natriuretic peptide OR B‐type natriuretic peptide OR brain natriuretic peptide OR BNP OR Amino terminal pro‐brain natriuretic peptide OR NT‐proBNP OR pro‐BNP OR proBNP OR NTproBNP OR N‐terminal pro‐brain natriuretic peptide OR N‐terminal pro‐BNP). The details of the search strategy used for this review were presented in (Additional file 1). In addition, the references in the included articles were screened to make sure that no relevant studies have been missed. To identify potentially relevant gray literature, we conducted searches in Google Scholar along with our searches in major databases. All the publications were uploaded to Endnote software for initial screening of titles and abstracts as well as to remove duplicates.

### 2.1 Inclusion and Exclusion Criteria

The selection process involved a two‐step approach. Initially, the titles and abstracts of all identified articles were screened by three independent reviewers to identify potentially relevant studies. Then, we did a full‐text review of the selected studies in detail to assess their eligibility according to our set inclusion criteria. The inclusion criteria for the articles in this review were cross‐sectional, case‐control, and cohort studies that supplied data on B‐type natriuretic peptides levels. Case reports, editorials, reviews, abstracts, and studies with incomplete data for the variables of interest were excluded. The initial inter‐reviewer agreement for study selection was calculated with a kappa statistic of 0.70.

Quality assessment and data extraction:

To evaluate the methodological rigor of the studies included for potential biases, critical appraisal checklists from the Joanna Briggs Institute were utilized (https://jbi.global/critical-appraisal-tools). These checklists facilitate assessment of the possibility of bias in study design, conduct, and data analysis. Data retrieved from every study included author, year, region, number of patients, age group of participants, and levels of B‐type natriuretic peptides for all groups. In cases where a study reported medians and interquartile ranges, mean and SD were estimated using the method described by Wan et al. [14]. In cases where a study provided means and SDs for multiple groups of asthmatic patients, we combined them into a single set of values for each study [15]. Any discrepancies during data extraction or quality assessment were resolved through discussion and consensus.

### 2.2 Statistical Analysis

The statistical analyses were performed by using Jamovi software (https://www.jamovi.org) to calculate the pooled standardized mean difference (SMD) and its 95% confidence intervals (CI). In addition, a meta‐analysis of correlation coefficient (*r*) was done to investigate the relationships between B‐type natriuretic peptides and various biomarkers in beta‐thalassemia patients. To obtain standardized effect sizes for each correlation coefficient, Fisher′s r‐to‐z transformation was applied by transforming the correlation coefficients into z‐scores, which are normally distributed, allowing for a more accurate estimation of the overall effect size and standard error. The overall pooled correlation coefficients were obtained by transforming the values back to the *r* scale using the inverse of the Fisher′s r‐to‐z transformation.

A random‐effects model, using DerSimonian–Laird method, was used for calculation of the pooled effect sizes to account for studies heterogeneity, which was evaluated using the I² statistic. To test for the presence of publication bias—a potential source of bias due to the tendency for studies with statistically significant results to be published more often—we performed statistical analyses using both Begg′s and Egger′s regression tests. In addition, funnel plots were visually checked to evaluate publication bias for analyses involving more than 10 studies [16, 17]. When there was evidence of a publication bias, the Duval and Tweedie trim‐and‐fill method was applied to account for potentially missing studies [18].

Due to the specific functionalities of OpenMeta‐Analyst software (version 10.10) (http://www.cebm.brown.edu/openmeta/) [19], leave‐one‐out sensitivity analyses was conducted, using this software, to evaluate the robustness of the meta‐analyses through assessing the influence of individual studies. In addition, meta‐regression was done to assess how specific study characteristics are associated with variation in effect sizes. The significance level for all analyses was set at 0.05.

## 3 Results

**Figure 1:**
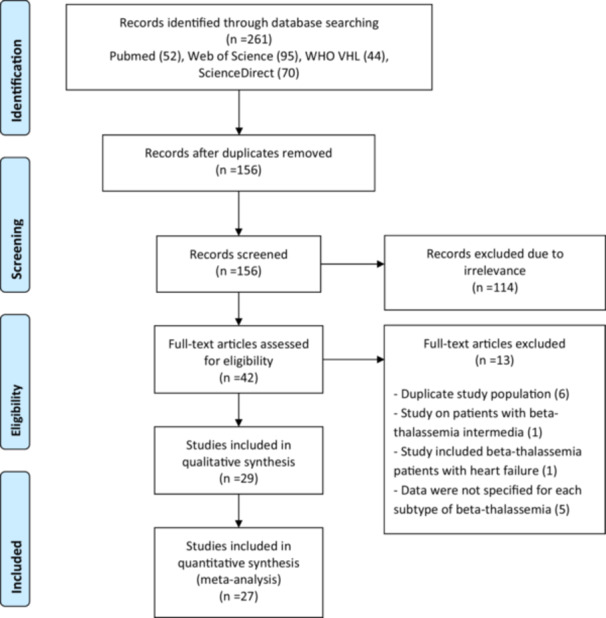
Flow chart for studies selection process.

Studies characteristics:

The schematic flow of the study identification and selection process is presented in Figure 1. The initial electronic database search yielded a total of 261 records. Following the removal of duplicate entries, 156 studies remained. These 156 studies underwent a thorough title and abstract screening process. During this stage, 114 studies were excluded for failing to meet our pre‐specified inclusion criteria. Full texts of the remaining 42 records were screened, leading to the exclusion of 13 records due to the reasons listed in Figure 1. Ultimately, a total of 29 studies were selected for the systematic review [8, 11, 20, 21, 22, 23, 24, 25, 26, 27, 28, 29, 30, 31, 32, 33, 34, 35, 36, 37, 38, 39, 40, 41, 42, 43, 44, 45, 46].

Most of the included studies focused on measuring NT‐proBNP, whereas a smaller group of six studies evaluated BNP, and only one study examined proBNP. Study publications ranged from 2006 to 2024, with a total of 1128 patients with beta‐thalassemia major. The main features of the selected studies, as well as risk of bias assessment, are presented in Table 1.

**Table 1:** Baseline characteristics of the studies included in the review.

Study	Year	Country	Age group	Age mean	No. of patients	B‐type natriuretic peptides	Quality assessment
Akpinar et al.	2007	Turkey	Adults	23.5 (4.5)	34	NT‐proBNP	6/8
Alizadeh et al.	2016	Iran	11–35 years	Mean of 17.9	50	NT‐proBNP	7/8
Ambrawati et al.	2016	Indonesia	Children	10.9 (2.16)	34	NT‐proBNP	6/8
Aygunes et al.	2022	Turkey	Children	82.2 months (59.1)	39	Pro‐BNP	6/8
Balkan et al.	2011	Turkey	Adults	24 (4.2)	49	NT‐proBNP	6/8
Cheema et al.	2012	Pakistan	6‐21 years	Median of 12	62	BNP	8/8
Chrysohoou et al.	2006	Greece	Adults	25.54 [6]		NT‐proBNP	6/8
Delaprota et al.	2013	Greece	Adults	34 (6.02)	187	NT‐proBNP	8/8
Deraz et al.	2020	Egypt	Children	10.5 (2.7)	50	NT‐proBNP	6/8
Elzemaiti et al.	2019	Egypt	16‐45 years	n/a	45	BNP	6/8
Garada et al.	2016	Bahrain	Adults	15.92 (8.92)	38	NT‐proBNP	6/8
Goudarzipour et al.	2017	Iran	Children	17.1 (5.3)	35	NT‐proBNP	7/8
Jewad et al.	2024	Iraq	All groups	12.2 (3.8**)**	46	NT‐proBNP	6/8
Kadhim et al.	2024	Iraq	Adults	22.67 (4.78)	30	BNP	6/8
Kanazirev et al.	2017	Bulgaria	Adults	31.6 (9.56)	87	NT‐proBNP	6/8
Karakas et al.	2022	Turkey	11 to 21 years	17.15 (3.13)	82	NT‐proBNP	8/8
Karamanou et al.	2013	Greece	Adults	36 (8.2)	88	NT‐proBNP	8/8
Kautsar et al.	2019	Indonesia	adolescent	14.01 (1.662)	68	NT‐proBNP	8/8
Kostopoulou et al.	2013	Greece	5 to 51 years	29 [11]	180	NT‐proBNP	8/8
Kremastinos et al.	2010	Greece	Adults	26.49 (11.55)	120	ProBNP & NT‐proBNP	8/8
Kurtoglu et al.	2017	Turkey	Adults	23.87 (2.76)	56	NT‐proBNP	7/8
Mehrzad et al.	2016	Iran	Adults	28 (6.4)	50	NT‐proBNP	8/8
Mohammed et al.	2019	Egypt	Children	12.15 (2.28)	70	BNP	7/8
Noori et al.	2019	Iran	Children	Mean of 16.7	114	NT‐proBNP	7/8
Özyörük et al.	2014	Turkey	Children	8.9 (3.5)	78	NT‐proBNP	6/8
Ragab et al.	2015	Egypt	4 to 20 years	10.9 (4.86)	45	BNP	8/8
Safniyat et al.	2020	Iran	8 to 40 years	26.3 (7.5)	123	NT‐proBNP	7/8
Singh et al.	2017	India	> 10 years	16.4 (5.1)	105	NT‐proBNP	7/8
Tanner et al.	2006	UK & Italy	Adults	30 (5.3)	167	NT‐proBNP	6/8

### 3.1 B‐Type Natriuretic Peptides Measurements

**Figure 2:**
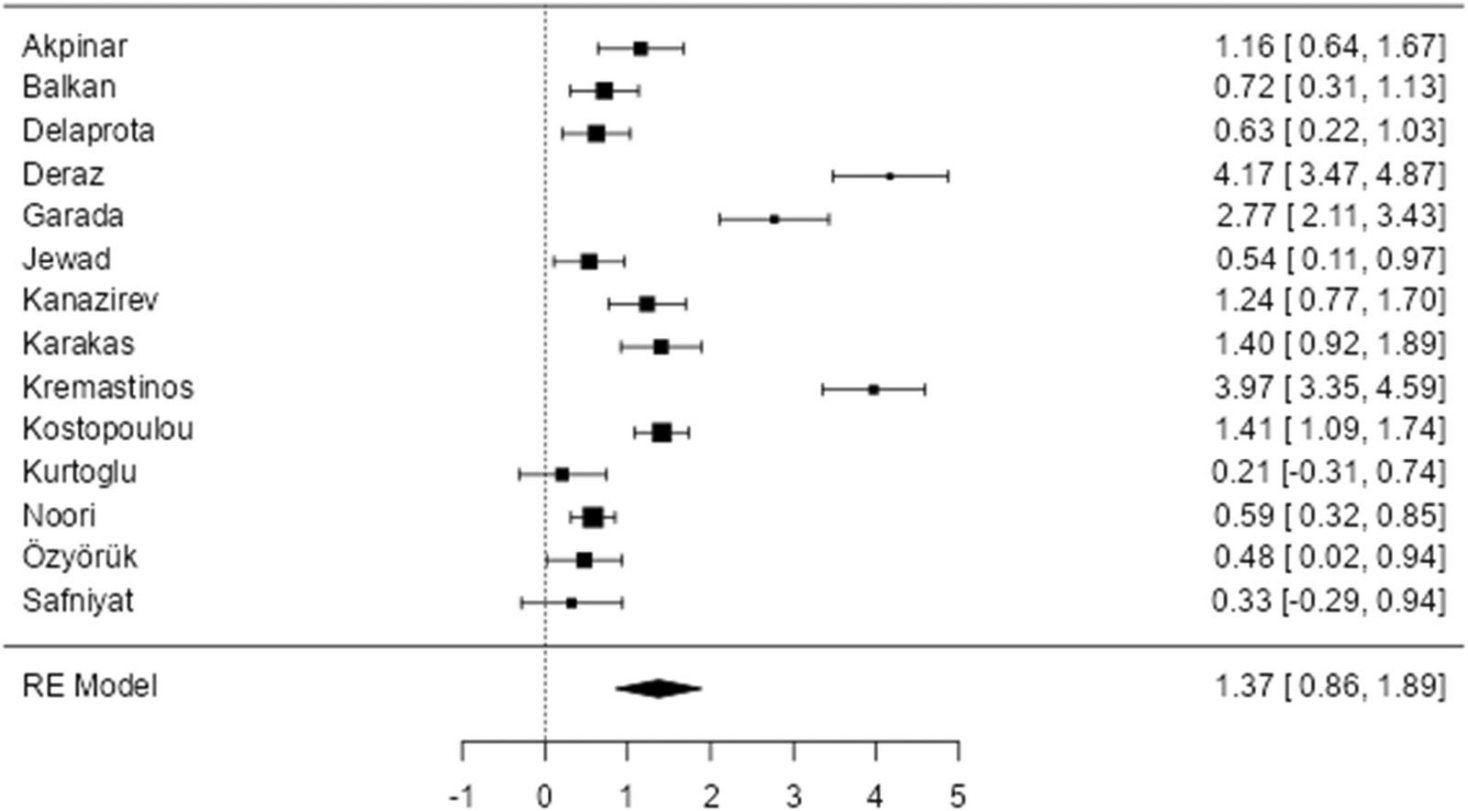
Forest plot for the pooled SMD of NT‐proBNP between patients with beta‐thalassemia major and healthy individuals.

**Figure 3:**
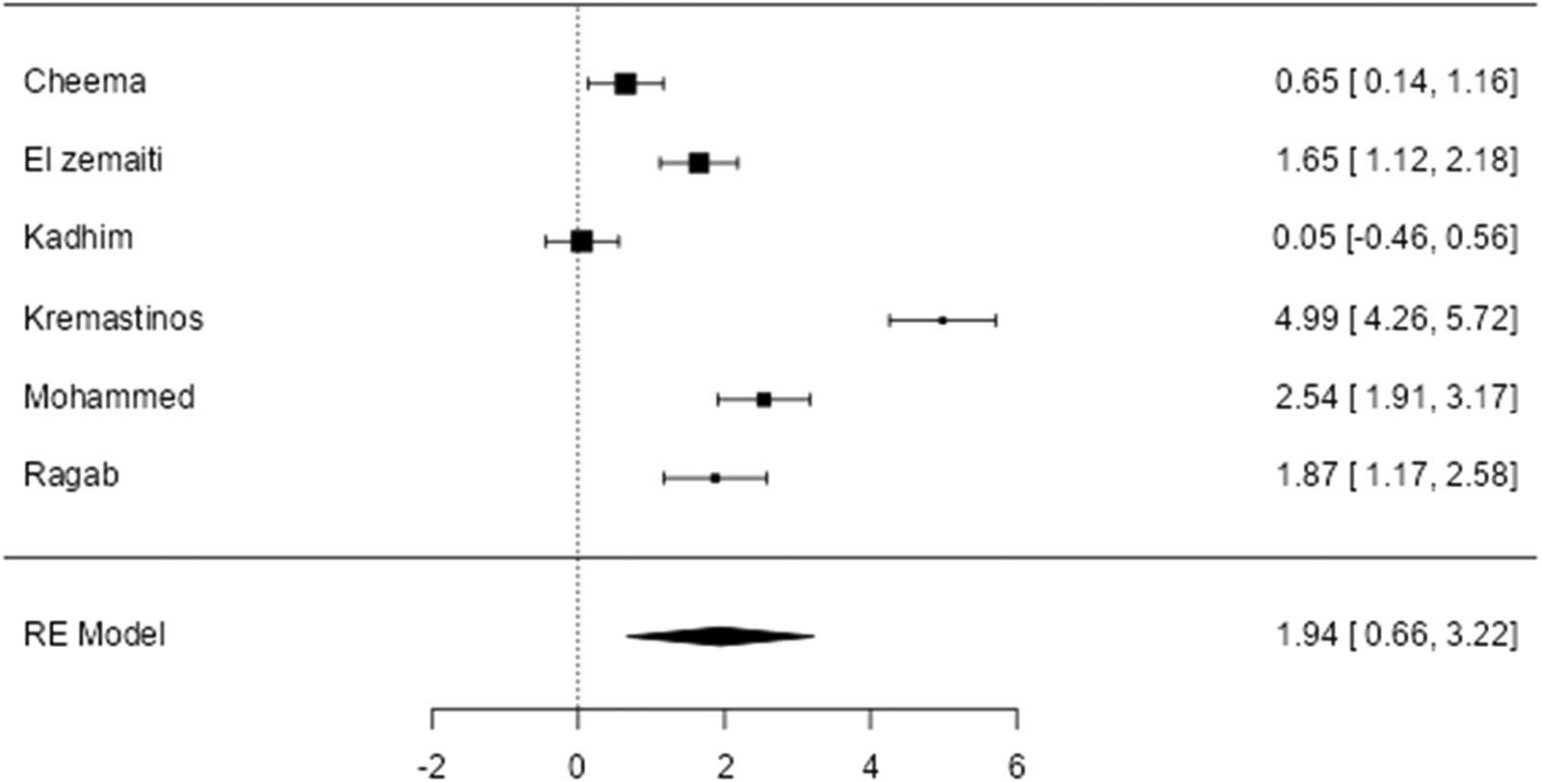
Forest plot for the pooled SMD of BNP between patients with beta‐thalassemia major and healthy individuals.

**Figure 4:**
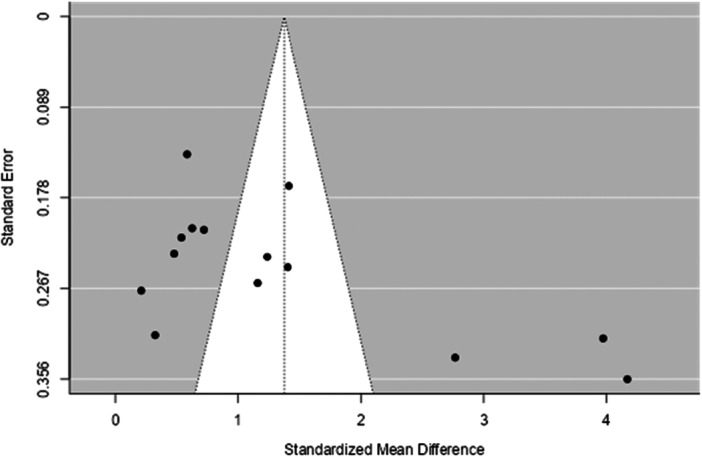
Funnel plot for the analysis of pooled SMD of NT‐proBNP between patients with beta‐thalassemia major and healthy individuals.

We conducted meta‐analyses to assess the difference in the mean levels of B‐type natriuretic peptides between patients and the healthy individuals (Figures 2, 3). The results showed that people with beta‐thalassemia major had significantly higher NT‐proBNP measurements than healthy individuals, with pooled effect size SMD = 1.37 (95% CI: 0.856–1.893, *p* < 0.001). Heterogeneity was high according to the I^2^ test (94.59%). The publication bias test was significant for Egger′s test (*p* = 0.001) but not for Begg′s test (*p* = 0.101) (Figure 4). The Duval and Tweedie trim‐and‐fill analysis did not identify any missing studies, an indication that the adjusted estimate was consistent with the original finding and unaffected by substantial publication bias.

There were six studies with explicit data to calculate the SMD of BNP estimates among patients with beta‐thalassemia major. The results showed that the patients had significantly higher BNP values than healthy individuals, with pooled effect size SMD = 1.94 (95% CI: 0.665–3.220, *p* = 0.003). Heterogeneity was high (96.47%) according to the I^2^ test. The publication bias test was significant for both Egger′s test (*p* < 0.001) and Begg′s test (*p* = 0.017). The Duval and Tweedie trim‐and‐fill analysis did not identify any missing studies, indicating that the adjusted estimate was consistent with the original finding and unaffected by substantial publication bias. Regarding Pro‐BNP, the findings from the only study that assessed proBNP showed a significant increase in proBNP amongst patients compared to healthy controls [23].

### 3.2 Moderators of Heterogeneity

The leave‐one‐out sensitivity analyses were conducted to assess the influence of individual studies on the pooled effect estimates. For NT‐proBNP, the results showed that all re‐analyses, where each study was removed one at a time, yielded a statistically significant pooled effect estimate (*p* < 0.001), indicating that the effect size from each individual study was always consistent and did not cause excessive change to the pooled estimates. Furthermore, meta‐regression analyses were conducted to analyze whether continuous variables (number of patients and publication year) affected the heterogeneity in the meta‐analysis. The results showed that neither number of patients (r = −0.004, *p* = 0.646) nor publication year (r = −0.032, *p* = 0.650) had a significant moderating effect on the outcome.

Regarding BNP, the leave‐one‐out sensitivity analysis showed that excluding each study individually did not substantially alter the overall statistical significance. However, it was noteworthy that removing the study conducted by Kremastinos et al. lead to in a much lower but still significant pooled estimate (SMD = 1.336, *p* = 0.003). The meta‐regression analyses showed that the outcome was affected by the number of patients enrolled in studies. Specifically, studies with larger sample sizes may have slightly larger effect sizes compared to studies with smaller sample sizes. However, this correlation was weak (r = 0.088, *p* < 0.001), indicating that the practical impact of sample size on the outcome may be small. On the other hands, the outcome was not significantly affected by publication year (r = −0.207, *p* = 0.063).

### 3.3 Associations Between B‐Type Natriuretic Peptides and Various Biomarkers

Multiple investigations have evaluated the association of B‐type natriuretic peptides with a broad range of markers of cardiac function and other biomarkers in beta‐thalassemia patients (Table 2). Some of the included studies evaluated diastolic function by measuring mitral valve inflow velocities (E and A) and mitral annular tissue velocities (E′ and A′) during early and late diastole. The most commonly identified factors were serum ferritin levels, patients age, and diastolic dysfunction based on the E/E′ ratio. Other echocardiographic parameters, like the E/A ratio, systolic wave velocity (S′), and deceleration time, also demonstrated significant correlations with B‐type natriuretic peptides in few studies. Conversely, several studies reported no significant association between B‐type natriuretic peptides and other variables such as cardiac MRI‐T2. Interestingly, some research assessed the diagnostic utility of B‐type natriuretic peptides by identifying cut‐off values that can accurately detect diastolic dysfunction based on E/E′ ratio. Despite considerable variation across studies, all of these cut‐off values showed high levels of sensitivity and specificity according to the ROC analyses (Table 2).

**Table 2:** Summary of findings related to associations between B‐type natriuretic peptides and various biomarkers.

Study	Main findings
Aessopos et al.	BNP was higher in thalassemia patients with heart failure. BNP was correlated with echocardiographic parameters (E and E/A ratio) in those with normal systolic function.
Akpinar et al.	NT‐proBNP was significantly associated with E/A ratio
Alizadeh et al.	NT‐proBNP was not correlated with age, sex, Hb level, or diastolic dysfunction
Ambrawati et al.	NT‐proBNP was correlated with vitamin D, but not with age, ferritin, and Hb levels
Aygunes et al.	ProBNP values were correlated with endocan and Asymmetric dimethyl arginine (ADMA)
Balkan et al.	NT‐proBNP was correlated with serum ferritin and echocardiographic parameters (E**′**, A**′**, and S**′**) but not with diastolic dysfunction (E/E′ ratio).
Cheema et al.	BNP was correlated with E/E′ ratio. The ROC curve analysis for BNP at a cut‐off value of 84.3 pg/mL was highly accurate (AUC = 0.86) in ruling out diastolic dysfunction (based on E/E′ ratio), with a sensitivity of 80% and specificity of 88%.
Chrysohoou et al.	BNP was significantly associated with echocardiographic parameter (E/A ratio, S′, and deceleration time)
Delaprota et al.	NT‐proBNP was correlated with age, cardiac iron concentration, hs‐CRP, and NTBI.
Deraz et al.	NT‐proBNP was correlated with serum ferritin and echocardiographic parameters (E, E/A ratio, E/E ratio, and S**′**).
Elzemaiti et al.	NT‐proBNP was correlated with serum ferritin and frequency of blood transfusion
Garada et al.	NT‐proBNP was correlated with the E/E′ ratio
Goudarzipour et al.	NT‐proBNP was correlated with heart failure and cardiac MRI‐T2
Jewad et al.	NT‐proBNP was correlated with age, GDF‐15, and renalase, but not with sex or serum ferritin
Kadhim et al.	No correlation between NT‐ProBNP and serum ferritin or troponin
Kanazirev et al.	NT‐proBNP was correlated with only one echocardiographic parameter (left atrial volume index) based on NT‐pro‐BNP > 125 pg/ml or less categorization
Karakas et al.	NT‐proBNP was not correlated with cardiac MRI‐T2
Karamanou et al.	BNP was higher in the diastolic dysfunction group (based on E/E′ ratio) without reaching statistical significance
Kautsar et al.	NT‐proBNP was not correlated with cardiac MRI‐T2
Kostopoulou et al.	NT‐proBNP was significantly associated with iron load, serum ferritin, diastolic dysfunction (E/E′ ratio) and AF.
Kremastinos et al.	Both BNP and NT‐proBNP were correlated with serum ferritin and E/E′ ratio. The ROC analysis for NT proBNP at a cut point of 49.2 pg/mL was highly accurate (AUC = 0.97) in ruling out diastolic dysfunction (based on E/E′ ratio) with a sensitivity of 93.7% and a specificity of 89.6%.
Kurtoglu et al.	No significant difference detected in NT‐proBNP between patients and controls.
Mehrzad et al.	NT‐proBNP was not correlated with cardiac MRI‐T2, age, chelation therapy, or serum ferritin.
Mohammed et al.	BNP was correlated with Impaired EF%. The ROC curve analysis for BNP at a cut‐off value of 31 pg/mL was highly accurate (AUC = 0.99) in predicting myocardial dysfunction, with a sensitivity of 93% and specificity of 100%.
Noori et al.	NT‐proBNP was correlated with echocardiographic parameters (EF, FS, QT) and BMI
Özyörük et al.	NT‐proBNP was correlated with age and echocardiographic parameters (right ventricular E/E′ ratio, left ventricle mass index, and left ventricular end diastole diameter)
Ragab et al.	BNP was correlated with serum ferritin and echocardiographic parameters (E/E′ ratio, E, and IRT). The ROC curve analysis for BNP at a cut‐off value of 28.5 pg/ml was highly accurate (AUC = 0.86) in ruling out diastolic dysfunction based on E/E′ ratio, with a sensitivity of 100%, specificity of 81.9%, negative predictive value of 100%, and positive predictive value of 75%.
Safniyat et al.	NT‐proBNP was correlated with LVIDd but not with cardiac MRI‐T2, E/E′ ratio, and serum ferritin in all patients groups (including beta‐thalassemia intermedia).
Singh et al.	NT‐proBNP was correlated with diastolic dysfunction, LVESD, E/E, and E/A ratios. The ROC curve analysis for NT‐proBNP at a cut‐off value of 81 pg/mL was highly accurate (AUC = 0.868) in predicting of diastolic dysfunction, with a sensitivity of 87.5% and specificity of 85.7%
Tanner et al.	BNP was correlated with cardiac iron overload (MRI‐T2)

Abbreviations: A: late trans‐mitral inflow velocity; A′: mitral annular velocity (late diastolic); E: early trans‐mitral inflow velocity; E′: mitral annular velocity (early diastolic); AF: atrial fibrillation; EF: ejection fraction; IRT: isovolumic relaxation time; LVIDd: left ventricular internal dimension in diastole; NTBI: non‐transferrin‐bound‐iron; hs‐CRP, high sensitivity C‐reactive protein; S′: mitral annular velocity (systolic).

Regarding meta‐analysis, few studies reported sufficient data for conducting meta‐analyses on correlations between NT‐proBNP and patients age, serum ferritin levels, E/E′ ratio, and cardiac MRI‐T2. An even smaller number of studies reported sufficient data for analyzing BNP correlations with serum ferritin levels.

The meta‐analyses results showed that NT‐proBNP was significantly correlated with serum ferritin levels (*r* = 0.471, 95% CI: 0.134–0.808; *p* = 0.006), E/E′ ratio (*r* = 0.528, 95% CI: 0.304–0.752; *p* < 0.001), and patients age (*r* = 0.259, 95% CI: 0.081–0.437; *p* = 0.004). However, NT‐proBNP was not significantly correlated with cardiac MRI‐T2 (*r* = ‐0.231, 95% CI: −0.747–0.285; *p* = 0.380). For BNP, the analysis revealed a significant correlation with serum ferritin levels (*r* = 0.347, 95% CI: 0.066–0.628; *p* = 0.015) (Additional file 2, Figure S1‐S5). Publication bias tests were not significant for most of these analyses (Table 3).

**Table 3:** Meta‐analyses of correlation between NT‐proBNP and various biomarkers among patients with beta‐thalassemia major.

B‐type natriuretic peptides	Outcome	No. of studies	Pooled correlation	Begg′s test (*p* value)	Egger′s test (*p* value)	I^2^ test
NT‐proBNP	Age	7	*r* = 0.259, *p* = 0.004	0.362	0.257	72.89%
	Ferritin	8	*r* = 0.471, *p* = 0.006	0.901	0.920	92.58%
	MRI‐T2	4	*r* = −0.231, *p* = 0.380	0.333	0.150	90.34%
	E/E ratio	5	*r* = 0.528, *p* < 0.001	0.083	0.002	72.25%
BNP	Ferritin	4	*r* = 0.347, *p* = 0.015	1.00	0.877	66.41%

## 4 Discussion

This systemic review assessed the differences in B‐type natriuretic peptide levels between beta‐thalassemia major patients and healthy individuals and evaluated correlations between B‐type natriuretic peptide and various biomarkers. Our review found that BNP and NT‐proBNP are significantly elevated in beta‐thalassemia major patients compared to healthy individuals, with SMD of 1.37 for NT‐proBNP and SMD of 1.94 for BNP. These very high levels of both natriuretic peptides indicate that patients with beta‐thalassemia major experience considerably elevated cardiac stress and volume overload compared to healthy individuals, making these markers important indicators of cardiac dysfunction in this population. It is noteworthy that these markers were measured even in the absence of overt heart failure in all of the studies reviewed, suggesting subclinical cardiac dysfunction.

The analysis showed significant correlations between NT‐proBNP and other markers of cardiac dysfunction in beta‐thalassemia patients including serum ferritin and the E/E′ ratio, a marker of diastolic dysfunction. The pathophysiology underlying these associations is likely multifactorial. Chronic anemia and increased cardiac output leads to sustained myocardial stress which leads to ventricular remodeling and increased natriuretic peptide levels. Oxidative stress and iron overload, common in beta‐thalassemia, leads to myocardial fibrosis and diastolic dysfunction which may explain the correlations between NT‐proBNP and E/E′ ratio [24, 47].

The correlation with serum ferritin suggest a complex relationship between chronic transfusion related iron deposition and myocardial dysfunction. Interestingly, we found no significant correlation between NT‐proBNP and cardiac MRI‐T2, which measures myocardial iron content. This suggests that these natriuretic peptides are reflecting functional impairment, like diastolic dysfunction, rather than the amount of myocardial iron, which can be captured by MRI‐T2 [36]. Moreover, the discordance between NT‐proBNP and cardiac MRI‐T2 suggests that iron deposition is a necessary but not sufficient condition for cardiac dysfunction and functional deterioration may result from a combination of iron toxicity, inflammation and endothelial dysfunction [48].

Prolonged exposure to these stressors can lead to myocardial fibrosis, impaired relaxation and increased left ventricular filling pressures [24]. The correlations between NT‐proBNP levels and diastolic dysfunction shows that cardiac involvement in beta‐thalassemia is likely insidious. Therefore, these markers play a key role in assessing early cardiac involvement in beta‐thalassemia patients and it can be an early warning sign of myocardial stress, allowing for intervention before irreversible damage occur.

Additionally, some of the included studies identified diagnostic cut‐off values for BNP and NT‐proBNP that were highly accurate in ruling out diastolic dysfunction. The reported thresholds for diagnosing diastolic dysfunction in beta‐thalassemia patients showed considerable variation across studies. Other studies have investigated cut‐off values for NT‐proBNP in detecting early or subclinical cardiac dysfunction in the general population, as well as in other high‐risk populations for heart failure [49, 50, 51]. When comparing these values to beta‐thalassemia patients, our systematic review identified significantly lower NT‐proBNP cut‐off values for diastolic dysfunction. This finding has important clinical implications, indicating the need for more cautious cardiac monitoring and potentially earlier intervention in beta‐thalassemia patients. However, establishment of reliable cut‐offs for this patients population remains a knowledge gap that should be addressed in future research.

Defining precise cut‐off values would makes these biomarkers even more useful as screening tools to reduce the need for expensive and less accessible imaging. By adding these biomarkers to clinical algorithms, we can move towards a more proactive and individualized approach to managing cardiac complications in beta‐thalassemia. Future studies should look at the longitudinal trend of these biomarkers in beta‐thalassemia patients and refine the cut‐offs for risk stratification. Also, investigating the interplay between these peptides and biomarkers of fibrosis and inflammation will also help us understand the pathophysiology of cardiac dysfunction in this patients population.

However, this review have some limitations. The high heterogeneity across studies likely due to different patient populations, study designs and methods may affect generalizability and should be taken into consideration when interpreting the pooled estimates. The inclusion of studies with both pediatric and adult populations may contributed to the observed heterogeneity. The inconsistency in reporting all clinically relevant outcomes across studies limited conducting further meta‐analyses on other variables relevant to the problem, especially the other markers of cardiac dysfunction. Future studies should aim for more methodological consistency and bigger sample sizes to strengthen the findings. By addressing these gaps, we can further refine the clinical use of natriuretic peptides and improve cardiac monitoring and outcomes in beta‐thalassemia patients. Finally, excluding non‐English studies could limit the breadth of conclusions.

## 5 Conclusion

This systematic review provides evidence that beta‐thalassemia major is associated with increased levels of B‐type natriuretic peptides, which reflects their increased cardiovascular risk. The significantly elevated levels of these molecules and their identified correlations emphasize clinical utility of them as markers of early myocardial stress and subclinical heart disease in beta‐thalassemia. In addition, the findings emphasize the need for regular assessment of in clinical practice to risk stratify patients and identify those at high risk for adverse cardiovascular outcomes, and to guide interventions to improve their outcomes and survival.

## Author Contributions

**Mohamed S K Salih:** conceptualization, methodology, writing – original draft, writing – review and editing, project administration. **Amna H Mohamed:** writing – original draft, writing – review and editing, investigation, data curation. **Elsara M A Mirghani:** investigation, data curation. **Mohammed Y K Makki:** data curation, investigation. **Ola A M Ahmed:** data curation, investigation. **Esraa T S Mohammed:** data curation, investigation. **Hana H Mohamed:** writing – review and editing, writing – original draft. **Nada Omar:** investigation, data curation. **Esraa M A Garalnabi:** investigation, data curation. **Sagad O O Mohamed:** conceptualization, writing – original draft, writing – review and editing, formal analysis, supervision, investigation, methodology, data curation, visualization, project administration.

## Conflicts of Interest

The authors declare that they have no competing interests.

## 1 Transparency Statement

The lead author Sagad O. O. Mohamed affirms that this manuscript is an honest, accurate, and transparent account of the study being reported; that no important aspects of the study have been omitted; and that any discrepancies from the study as planned (and, if relevant, registered) have been explained.

## Supporting information

Additional file 1.

Additional file 2.

## Data Availability

Data sharing not applicable to this article as no datasets were generated or analyzed during the current study.

## References

[1] A. T. Taher , K. M. Musallam , and M. D. Cappellini , “β‐Thalassemias.” in N Engl J Med, eds. D. L. Longo , 2021 Feb 25. 384, 727–743. 8. 33626255 10.1056/NEJMra2021838

[2] M. Sanchez‐Villalobos , M. Blanquer , J. M. Moraleda , E. J. Salido , and A. B. Perez‐Oliva , “New Insights Into Pathophysiology of β‐Thalassemia,” Frontiers in Medicine 9 (April 2022): 880752. 35492364 10.3389/fmed.2022.880752 PMC9041707

[3] R. Origa , “β‐Thalassemia,” Genetics in Medicine 19, no. 6 (June 2017): 609–619. 27811859 10.1038/gim.2016.173

[4] Y. M. Lee , S. Y. Chuang , S. F. Wang , Y. T. Lin , and Y. M. A. Chen , “Epidemiology of Human Herpesvirus Type 8 and Parvovirus B19 Infections and Their Association With HIV‐1 Among Men Who Have Sex With Men and Injection Drug Users in Taiwan,” Journal of Microbiology, Immunology and Infection 47, no. 3 (June 2014): 233–238. 10.1016/j.jmii.2013.01.006 23465711

[5] C. Hershko , “Pathogenesis and Management of Iron Toxicity in Thalassemia,” Annals of the New York Academy of Sciences 1202, no. 1 (August 2010): 1–9. 20712765 10.1111/j.1749-6632.2010.05544.x

[6] F. Koohi , T. Kazemi , and E. Miri‐Moghaddam , “Cardiac Complications and Iron Overload in Beta Thalassemia Major Patients—A Systematic Review and Meta‐Analysis,” Annals of Hematology 98, no. 6 (June 2019): 1323–1331. 30729283 10.1007/s00277-019-03618-w

[7] S. Lekawanvijit and N. Chattipakorn , “Iron Overload Thalassemic Cardiomyopathy: Iron Status Assessment and Mechanisms of Mechanical and Electrical Disturbance Due to Iron Toxicity,” Canadian Journal of Cardiology 25, no. 4 (2009): 213–218. 19340344 10.1016/s0828-282x(09)70064-9 PMC2706758

[8] A. Kautsar , N. Advani , and M. Andriastuti , “N‐Terminal‐Pro‐B‐Type Natriuretic Peptide Levels and Cardiac Hemosiderosis in Adolescent β‐thalassemia Major Patients,” Annals of Pediatric Cardiology 12, no. 1 (2019): 32. 30745767 10.4103/apc.APC_49_18 PMC6343373

[9] S. M. Shehata , M. I. Amin , and E. S. H. Zidan , “MRI Evaluation of Hepatic and Cardiac Iron Burden in Pediatric Thalassemia Major Patients: Spectrum of Findings by T2*,” Egyptian Journal of Radiology and Nuclear Medicine 50, no. 1 (December 2019): 68.

[10] A. Khaled , D. A. Ezzat , H. A. Salem , H. M. Seif , and H. Rabee , “Effective Method of Evaluating Myocardial Iron Concentration in Pediatric Patients With Thalassemia Major,” Journal of Blood Medicine 10 (July 2019): 227–233. 31372080 10.2147/JBM.S204848 PMC6636185

[11] S. E. Deraz , S. A. A. El Naby , and A. A. Mahmoud , “Assessment of Ventricular Dysfunction in Egyptian Children with Beta‐Thalassemia Major,” Hematology/Oncology and Stem Cell Therapy 14, no. 3 (July 2021): 206–213. 32758485 10.1016/j.hemonc.2020.07.003

[12] A. Bayes‐Genis , K. F. Docherty , M. C. Petrie , et al., “Practical Algorithms for Early Diagnosis of Heart Failure and Heart Stress Using NT‐ProBNP: A Clinical Consensus Statement From the Heart Failure Association of the ESC,” European Journal of Heart Failure 25, no. 11 (November 2023): 1891–1898. 37712339 10.1002/ejhf.3036

[13] A. Liberati , D. G. Altman , J. Tetzlaff , et al., “The PRISMA Statement for Reporting Systematic Reviews and Meta‐Analyses of Studies That Evaluate Health Care Interventions: Explanation and Elaboration,” Journal of Clinical Epidemiology 62, no. 10 (October 2009): e1–e34. 19631507 10.1016/j.jclinepi.2009.06.006

[14] X. Wan , W. Wang , J. Liu , and T. Tong , “Estimating the Sample Mean and Standard Deviation From the Sample Size, Median, Range And/Or Interquartile Range,” BMC Medical Research Methodology 14, no. 1 (December 2014): 135. 25524443 10.1186/1471-2288-14-135 PMC4383202

[15] A. Abbas , M. T. Hefnawy , and A. Negida , “Meta‐Analysis Accelerator: A Comprehensive Tool for Statistical Data Conversion In Systematic Reviews with Meta‐Analysis,” BMC Medical Research Methodology 24, no. 1 (October 2024): 243. 39425031 10.1186/s12874-024-02356-6 PMC11487830

[16] C. B. Begg and M. Mazumdar , “Operating Characteristics of a Rank Correlation Test for Publication Bias,” Biometrics 50, no. 4 (December 1994): 1088. 7786990

[17] M. Borenstein , L. V. Hedges , J. P. T. Higgins , and H. R. Rothstein , “A Basic Introduction to Fixed‐Effect and Random‐Effects Models for Meta‐Analysis,” Research Synthesis Methods 1, no. 2 (April 2010): 97–111. 26061376 10.1002/jrsm.12

[18] S. Duval and R. Tweedie , “Trim and Fill: A Simple Funnel‐Plot–Based Method of Testing and Adjusting for Publication Bias in Meta‐Analysis,” Biometrics 56, no. 2 (June 2000): 455–463. 10877304 10.1111/j.0006-341x.2000.00455.x

[19] B. C. Wallace , I. J. Dahabreh , T. A. Trikalinos , J. Lau , P. Trow , and C. H. Schmid , “Closing the Gap Between Methodologists and End‐Users: R as a Computational Back‐End,” J Stat Softw [Internet] 49, no. 5 (2012): 1, http://www.jstatsoft.org/v49/i05/.

[20] O. Akpinar , E. Acartürk , M. Kanadaşi , Ç. Ünsal , and F. Başlamişli , “Tissue Doppler Imaging and NT‐ProBNP Levels Show the Early Impairment of Ventricular Function in Patients With ß‐Thalassaemia Major,” Acta Cardiologica 62, no. 3 (2007): 221–223. 10.2143/ac.62.3.2020809 17608095

[21] B. Alizadeh , Z. Badiee , M. Mahmoudi , and M. Mohajery , “Evaluating the Correlation Between Serum NT‐proBNP Level and Diastolic Dysfunction Severity in Beta‐ Thalassemia Major Patients.” Journal of Tehran Heart Center 11, no. 2 (2016): 68–72. 27928257 PMC5027163

[22] P. Ambrosino , G. Marcuccio , F. Manzo , C. Mancusi , C. Merola , and M. Maniscalco , “The Clinical Application of Established and Emerging Biomarkers for Chronic Respiratory Diseases,” Journal of Clinical Medicine 12, no. 19 (October 2023): 6125. 37834769 10.3390/jcm12196125 PMC10573548

[23] U. Aygüneş , Ü. Can , M. Doğan , M. Keçeli , and H. K. Eker The Effect of Plasma Endocan and Asymmetric Dimethyl Arginine Levels on Endothelial and Cardiac Functions in Children With Beta Thalassemia Major [Internet] 2024.

[24] C. Balkan , S. Y. Tuluce , and G. Basol , et al., “Relation Between NT‐ProBNP Levels, Iron Overload, and Early Stage of Myocardial Dysfunction in β‐Thalassemia Major Patients,” Echocardiography 29, no. 3 (March 2012): 318–325. 22066516 10.1111/j.1540-8175.2011.01584.x

[25] A. N. Cheema , D. A. Khan , and F. Tuyyab , “Early Detection of Cardiac Dysfunction by BNP in Beta‐Thalassaemia Major Patients,” Acta Cardiologica 67, no. 3 (June 2012): 331–335. 22870742 10.1080/ac.67.3.2160723

[26] C. Chrysohoou , D. B. Panagiotakos , C. Pitsavos , K. Kosma , J. Barbetseas , M. Karagiorga , et al., “Distribution of Serum Lipids and Lipoproteins in Patients With Beta Thalassaemia Major; An Epidemiological Study in Young Adults From Greece,” Lipids in Health and Disease 3 (2004): 3. 15023232 10.1186/1476-511X-3-3 PMC385250

[27] P. Delaporta , A. Kattamis , and F. Apostolakou , “Correlation of NT‐ProBNP Levels and Cardiac Iron Concentration in Patients With Transfusion‐Dependent Thalassemia Major,” Blood Cells, Molecules, and Diseases 50, no. 1 (January 2013): 20–24. 10.1016/j.bcmd.2012.09.002 23017692

[28] M. El Zimaity , H. Abdelbary , and H. Mohamed , “Brain Natriuretic Peptide as a Sensitive Biomarker for Early Detection of Cardiac Affection in Adult Egyptian Patients With β‐Thalassemia,” Egyptian Journal of Haematology 44, no. 1 (2019): 34.

[29] T. Garadah , A. A. Jaradat , M. AlAlawi , A. B. Hassan , and R. Sequeira , “Pain Frequency, Severity and QT Dispersion in Adult Patients With Sickle Cell Anemia: Correlation With Inflammatory Markers,” Journal of Blood Medicine 7 (October 2016): 255–261. 27843377 10.2147/JBM.S114585 PMC5098784

[30] K. Goudarzipour , P. Alizadeh , H. H. Tavassol , R. Kazemi , P. Eshghi , and S. Mojtahedzadeh , “A Comparison Between MRIT2 and NT‐ProBNP in Early Detection of Heart Diseases in Thalassemia Major Patients: A Cross‐Sectional Study,” Indian Journal of Hematology and Blood Transfusion 33, no. 4 (December 2017): 541–544. 29075066 10.1007/s12288-017-0797-9 PMC5640548

[31] A. J. Kadhim , H. D. El‐Yaseen , and A. M. Jawad , “The Level of Heart‐Type Fatty Acid Binding Protein (H‐FABP) as Risk Marker for Cardiac Dysfunction Among Some Beta‐Thalassemia Major Patients in Baghdad City‐Iraq,” Pak J Life Soc Sci PJLSS [Internet] 22, no. 1 (2024): 3699–3706.

[32] H. Karakaş , A. G. Eroğlu , N. G. Akyel , et al., “Can Biomarkers Predict Myocardial Iron Overload in Children With Thalassemia Major?,” Cardiology in the Young 33, no. 11 (November 2023): 2203–2208. 36606531 10.1017/S1047951122004206

[33] B. Kanazirev , M. Dimova , V. Kaleva , et al. Early Identification of Heart Failure in Patients With Thalassemia Major by NT‐pro‐BNP Examination. Correlation With Echocardiographic Parameters of Morphology and Function. Int J Med Health Res.

[34] A. G. Karamanou , E. S. Hamodraka , S. C. Vrakas , I. Paraskevaidis , I. Lekakis , and D. D. T. Kremastinos , “Assessment of Left Ventricular and Atrial Diastolic Function Using Two‐Dimensional (2 D) Strain Imaging in Patients With β‐thalassemia Major,” European Journal of Haematology 92, no. 1 (January 2014): 59–65. 24118422 10.1111/ejh.12209

[35] A. G. Kostopoulou , D. P. Tsiapras , A. S. Chaidaroglou , D. E. De giannis , D. Farmakis , and D. D. T. Kremastinos , “The Pathophysiological Relationship and Clinical Significance of Left Atrial Function and Left Ventricular Diastolic Dysfunction in β‐thalassemia Major,” American Journal of Hematology 89, no. 1 (January 2014): 13–18. 24038100 10.1002/ajh.23581

[36] D. T. Kremastinos , E. Hamodraka , J. Parissis , D. Tsiapras , K. Dima , and A. Maisel , “Predictive Value of B‐Type Natriuretic Peptides in Detecting Latent Left Ventricular Diastolic Dysfunction in β‐thalassemia Major,” American Heart Journal 159, no. 1 (January 2010): 68–74. 20102869 10.1016/j.ahj.2009.10.025

[37] V. Mehrzad , A. Sajjadieh Khajouei , and E. Fahami , “Correlation of N‐Terminal Pro‐B‐Type Natriuretic Peptide Levels and Cardiac Magnetic Resonance Imaging T2* in Patients With ß‐Thalassaemia Major,” Blood Transfus [Internet] 14, no. 6 (2016): 516–520, 10.2450/2016.0120-15. 27136436 PMC5111375

[38] A. G. Mohammed and A. A. Elmalah . "Biomarkers of Myocardial Dysfunction in Children With B‐Thalassemia Major: Controlled Study" Saudi Journal of Biomedical Research 4, no. 3 (2019): 81–87, 10.21276/sjbr.2019.4.3.1.

[39] N. M. Noori , M. Nakhaee‐Moghadam , A. Teimouri , and A. R. Teimouri , “TNF‐α, Interleukin‐6, NT‐PRO BNP Correlation With Echocardiography Findings in Patients With Thalassemia.” Pakistan Heart Journal 52, No. 1 (2019).

[40] S. Ragab , “The Diagnostic Value of Pulsed Wave Tissue Doppler Imaging in Asymptomatic Beta‐ Thalassemia Major Children and Young Adults; Relation to Chemical Biomarkers of Left Ventricular Function and Iron Overload,” Mediterranean Journal of Hematology and Infectious Diseases 7 (August 2015): e2015051. 26401240 10.4084/MJHID.2015.051 PMC4560260

[41] S. Safniyat , N. Shakibazad , S. Haghpanah , et al., “Parameters of Tissue Iron Overload and Cardiac Function in Patients With Thalassemia Major and Intermedia,” Acta Haematologica Polonica 51, no. 2 (June 2020): 95–101.

[42] M. Singh , R. Kumar , S. Tewari , and S. Agarwal , “Determining NT‐ProBNP Levels With Diastolic Dysfunction in Thalassemia Major Patients,” Journal of Pediatric Genetics 06, no. 04 (December 2017): 222–226. 10.1055/s-0037-1603193 PMC5683953 29142764

[43] M. Tanner , R. Galanello , C. Dessi , et al., “Myocardial Iron Loading in Patients With Thalassemia Major on Deferoxamine Chelation,” Journal of Cardiovascular Magnetic Resonance 8, no. 3 (July 2006): 543–547. 16755844 10.1080/10976640600698155

[44] A. M. Jewad and A. J. Shwayel , “Evaluation of Some Nonroutine Cardiac Biomarkers Among Adults and Children With Beta‐Thalassemia Major,” Laboratory Medicine 55 (2024): 559–565. 38417033 10.1093/labmed/lmae007

[45] D. Özyörük , T. Öner , Y. Oymak , and H. T. Çelik , “Comparison of Doppler Echocardiographic and Tissue Doppler Velocity Data in Beta‐Thalassaemia Major With High and Normal NT‐ProBNP Levels of Children in the South‐East Region of Turkey,” Translational Pediatrics 3, no. 4 (2014): 287–292. 26835348 10.3978/j.issn.2224-4336.2014.06.02 PMC4728833

[46] A. U. Kurtoğlu , V. Karakuş , E. Kurtoğlu , and S. Bozkurt , “NT‐ProBNP Levels in β‐thalassemia Major Patients Without Cardiac Hemosiderosis,” Turkish Journal of Biochemistry 42, no. 1 (2017): 71–75.

[47] J. C. Wood , “Cardiac Complications in Thalassemia Throughout the Lifespan: Victories and Challenges,” Annals of the New York Academy of Sciences 1530, no. 1 (December 2023): 64–73, 10.1111/nyas.15078. 37902424 PMC10841366

[48] N. Akiki , M. H. Hodroj , R. Bou‐Fakhredin , K. Matli , and A. T. Taher , “Cardiovascular Complications in β‐Thalassemia: Getting to the Heart of It,” Thalassemia Reports 13, no. 1 (January 2023): 38–50.

[49] M. Averina , M. Stylidis , J. Brox , and H. Schirmer , “NT‐ProBNP and High‐Sensitivity Troponin T as Screening Tests for Subclinical Chronic Heart Failure in a General Population,” ESC Heart Failure 9, no. 3 (June 2022): 1954–1962. 35322586 10.1002/ehf2.13906 PMC9065856

[50] I. Betti , G. Castelli , A. Barchielli , et al., “The Role of N‐Terminal PRO‐Brain Natriuretic Peptide and Echocardiography for Screening Asymptomatic Left Ventricular Dysfunction in a Population at High Risk for Heart Failure. The PROBE‐HF Study,” Journal of Cardiac Failure 15, no. 5 (June 2009): 377–384. 19477397 10.1016/j.cardfail.2008.12.002

[51] C. Tschöpe , M. Kašner , D. Westermann , R. Gaub , W. C. Poller , and H. P. Schultheiss , “The Role of NT‐proBNP in the Diagnostics of Isolated Diastolic Dysfunction: Correlation With Echocardiographic and Invasive Measurements,” European Heart Journal 26, no. 21 (November 2005): 2277–2284. 16014646 10.1093/eurheartj/ehi406

