# Supplementary material for: B‐Type Natriuretic Peptides Levels in Patients With Beta‐Thalassemia Major and Correlations With Biomarkers: A Systematic Review and Meta‐Analysis
Source: Health Sci Rep. 2025 Nov 9;8(11):e71493. doi: 10.1002/hsr2.71493 (PMC12598194; doi:10.1002/hsr2.71493)
Supplement: Supplementary file 1 — Additional file 1. [file HSR2-8-e71493-s002.docx]

**Search strategy**

| **Database** | **Search term** |
| --- | --- |
| **Pubmed** | (thalassemia[Text Word] OR "beta-thalassemia"[Text Word] OR β-thalassemia[Text Word]) AND (Natriuretic peptide[Text Word] OR B‐type natriuretic peptide[Text Word] OR brain natriuretic peptide[Text Word] OR BNP[Text Word] OR Amino terminal pro-brain natriuretic peptide[Text Word] OR NT-proBNP[Text Word] OR pro-BNP[Text Word] OR proBNP[Text Word] OR NTproBNP[Text Word] OR N-terminal pro-brain natriuretic peptide[Text Word] OR N-terminal pro-BNP[Text Word]) |
| **Web of science** | **#1**: TS=(thalassemia OR "beta-thalassemia" OR β-thalassemia)  **#2:** TS**=**(Natriuretic peptide OR B‐type natriuretic peptide OR brain natriuretic peptide OR BNP OR Amino terminal pro-brain natriuretic peptide OR NT-proBNP OR pro-BNP OR proBNP OR NTproBNP OR N-terminal pro-brain natriuretic peptide OR N-terminal pro-BNP)  **#3**= (#1 AND #2) |
| **WHO VHL** | In titles, abstracts, and keywords:  (thalassemia OR "beta-thalassemia" OR β-thalassemia) AND (Natriuretic peptide OR B‐type natriuretic peptide OR brain natriuretic peptide OR BNP OR Amino terminal pro-brain natriuretic peptide OR NT-proBNP OR pro-BNP OR proBNP OR NTproBNP OR N-terminal pro-brain natriuretic peptide OR N-terminal pro-BNP) |
| **ScienceDirect** | In titles, abstracts, and keywords:  (thalassemia OR "beta-thalassemia" OR β-thalassemia) AND (Natriuretic peptide OR B‐type natriuretic peptide OR brain natriuretic peptide OR BNP OR Amino terminal pro-brain natriuretic peptide OR NT-proBNP OR pro-BNP OR proBNP OR NTproBNP OR N-terminal pro-brain natriuretic peptide OR N-terminal pro-BNP) |
