# Supplementary material for: B‐Type Natriuretic Peptides Levels in Patients With Beta‐Thalassemia Major and Correlations With Biomarkers: A Systematic Review and Meta‐Analysis
Source: Health Sci Rep. 2025 Nov 9;8(11):e71493. doi: 10.1002/hsr2.71493 (PMC12598194; doi:10.1002/hsr2.71493)
Supplement: Supplementary file 2 — Additional file 2. [file HSR2-8-e71493-s001.docx]

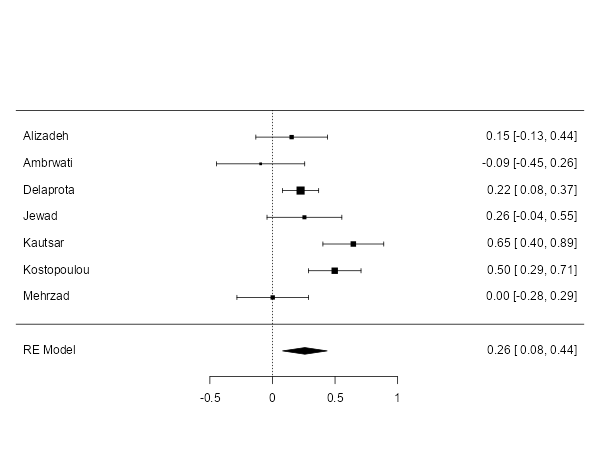


**Figure S1. Forest plot for the pooled correlation of NT-proBNP with age among patients with beta-thalassemia major**


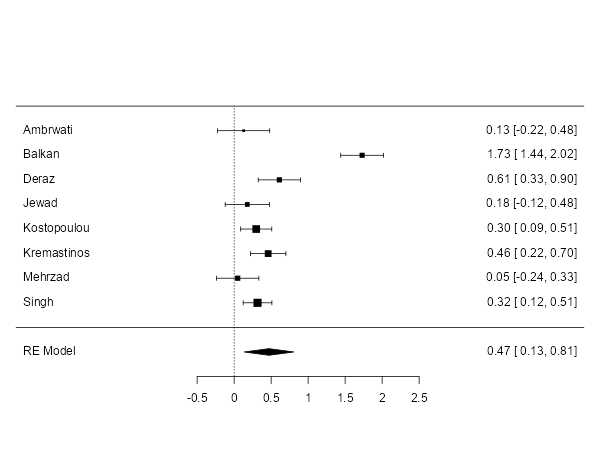


**Figure S2. Forest plot for the pooled correlation of NT-proBNP with serum ferritin among patients with beta-thalassemia major**


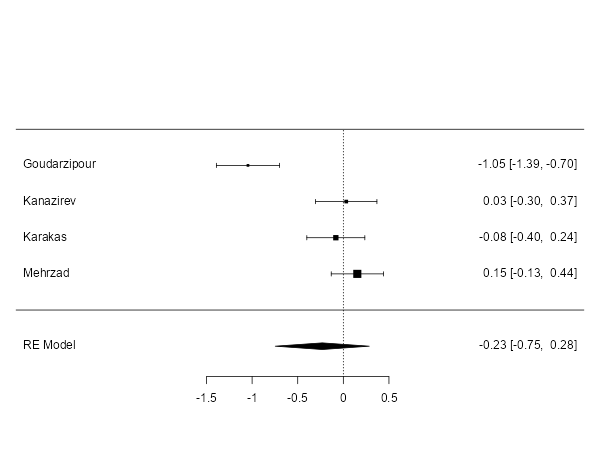


**Figure S3. Forest plot for the pooled correlation of NT-proBNP with cardiac MRI-T2 among patients with beta-thalassemia major**


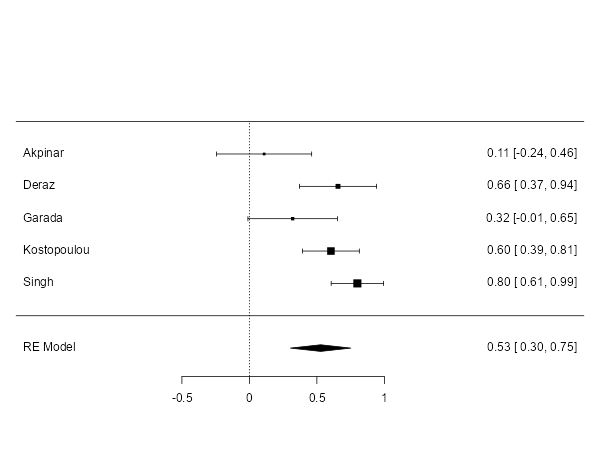


**Figure S4. Forest plot for the pooled correlation of NT-proBNP with E/E' ratio among patients with beta-thalassemia major**


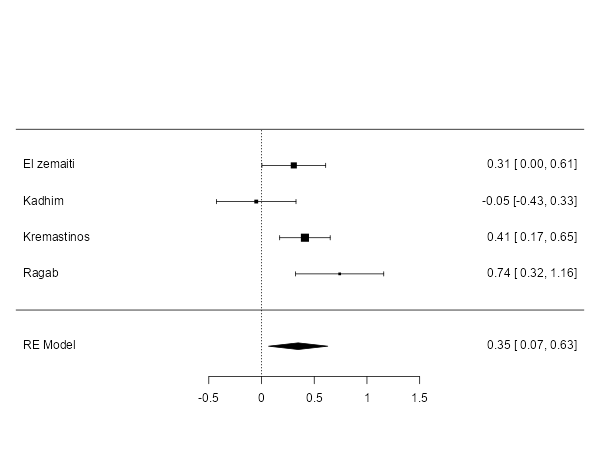


**Figure S5. Forest plot for the pooled correlation of BNP with serum ferritin among patients with beta-thalassemia major**
